# Integrating Metabolomics and Proteomics Technologies Provides Insights into the Flavor Precursor Changes at Different Maturity Stages of Arabica Coffee Cherries

**DOI:** 10.3390/foods12071432

**Published:** 2023-03-28

**Authors:** Zelin Li, Bin Zhou, Tingting Zheng, Chunyan Zhao, Xiaojing Shen, Xuefeng Wang, Minghua Qiu, Jiangping Fan

**Affiliations:** 1 College of Food Science and Technology, Yunnan Agricultural University, Kunming 650201, China; 2 State Key Laboratory of Phytochemistry and Plant Resources in West China, Kunming Institute of Botany, Chinese Academy of Sciences, Kunming 650201, China

**Keywords:** Arabica coffee, ripening, flavor precursors, metabolomics, proteomics

## Abstract

The metabolic modulation of major flavor precursors during coffee cherry ripening is critical for the characteristic coffee flavor formation. However, the formation mechanism of flavor precursors during coffee cherry ripening remains unknown. In the present study, a colorimeter was employed to distinguish different maturity stages of coffee cherry based on the coffee cherry skin colors, and proteomics and metabolomics profiles were integrated to comprehensively investigate the flavor precursor dynamics involved in Arabica coffee cherry ripening. The data obtained in the present study provide an integral view of the critical pathways involved in flavor precursor changes during coffee cherry ripening. Moreover, the contributions of critical events in regulating the development of flavor precursors during the four ripening stages of coffee cherries, including the biosynthesis and metabolism pathways of organic acids, amino acids, flavonoids, and sugars, are discussed. Overall, a total of 456 difference express metabolites were selected, and they were identified as being concentrated in the four maturity stages of coffee cherries; furthermore, 76 crucial enzymes from the biosynthesis and metabolism of sugars, organic acids, amino acids, and flavonoids contributed to flavor precursor formation. Among these enzymes, 45 difference express proteins that could regulate 40 primary amino acids and organic acids flavor precursors were confirmed. This confirmation indicates that the metabolic pathways of amino acids and organic acids played a significant role in the flavor formation of Arabica coffee cherries during ripening. These results provide new insights into the protease modulation of flavor precursor changes in Arabica coffee cherry ripening.

## 1. Introduction

*Coffea arabica* L., a genus of the family *Rubiaceae*, accounts for 60% of global coffee production, owing to the unique taste and pleasant aroma of the beverage [1]. The complex and distinct flavor of coffee beverages is mainly dependent not only on the roasting process conditions of coffee beans but also on the chemical compounds present in the fresh coffee cherry, which act as aroma precursors [2]. Primary coffee cherry components include carbohydrates, proteins, amino acids, trigonelline, caffeine, lipids, organic acids, flavonoid, and glycosides [3]. Among the pool of green coffee constituents, however, carbohydrates (sugars), proteins, acids fraction, flavonoids, and lipids have been suggested to be the principal flavor precursors [4]. Carbohydrates represent 40–65% of dry green coffee beans, and Arabica coffee contains more sugars than Robusta coffee [5]. During the roasting process, 12–24% of the sugars are degraded during light roasting, while 35–40% of sugars are degraded upon dark roasting [6], contributing to the caramel aroma, black color, and characteristic taste via the Maillard reaction and caramelization. The acid fractions, such as chlorogenic acids, could contribute to bitter and tart flavors, and amino acids serve as precursors for the Maillard reaction, which generates sweet, flower-like, and fruity flavors [7]. Wang et al. [8] found that 2-acetylthiazole, which contributes to fried chestnut, toasted cereal, roast meat, and nut flavors, could be formed by the reaction of D-glucose with L-cysteine. However, previous research mainly focused on the content of a single flavor precursor or the heat reaction changes in precursor compounds during coffee processing. To date, the integrated analysis of flavor precursors which might contribute to Arabica coffee cherry has not been reported.

In recent years, omics technology has been considered an effective way to reveal the relationship between metabolites, proteins, and genes [9]. The high-quality coffee genome-wide sequence has made it possible to apply transcriptomic and proteomic analysis techniques to detect the gene and protein compositions in coffee cherries [10,11], while applying transcriptomics alone could not accurately predict the protein abundance and protease activity [11]. Combined metabolomics and proteomics could be used for qualitative and quantitative analyses of low-molecular-weight metabolites and high-molecular-weight proteins, and the dynamic state of the cell, respectively [12]. To date, most proteomics and metabolomics profiling on fruits have mainly concentrated on exploring their developmental ripening process, carbohydrate metabolism, and stress response [12,13,14,15]. There is little research on applying metabolomics and proteomics to extend the flavor precursors changing coffee cherry ripening. Moreover, whether the flavor precursors change in metabolism pathways and during protease metabolism between coffee cherries and ripening behaviors remains to be studied. Thus, proteomics and metabolomics profiling were employed to understand the dynamic changes in metabolites and proteins that regulate the content of flavor precursors during the ripening of Arabica coffee cherries. The present study aims to present a dynamic map of Arabica coffee cherry ripening, concentrating on the processes of sugar, organic acids, and amino acids and the biosynthesis and metabolism of flavonoids to provide insight into the modulation of flavor precursor formation. These data would provide valuable information for further studies on the functions of these flavor precursors and influence coffee bean quality. The results would be interesting in understanding the flavor precursor formation in Arabica coffee cherry and in providing novel insights into the modulation of Arabica coffee cherry ripening.

## 2. Materials and Methods

### 2.1. Materials

Fresh *Coffea Arabica* L. cherries were used in this study and picked at an altitude of 1040 m in December 2020 from Baoshan City, Yunnan Province, China (25°1′13″ N, 98°49′30″ E). The coffee cherries sampled did not contain pests or carry diseases. The voucher specimens Coffea Arabica L. cherries (No. ACCS1-4) were obtained from the College of Food Science and Technology, Yunnan Agricultural University. The coffee cherries were divided into four groups (A, B, C, and D) based on their maturity level using color measurements (*n* = 20 per group). The samples from 20 cherries per group in one biological replicate were immediately frozen in liquid nitrogen for untargeted metabolomics (*n* = 6 per group) and proteomic analysis (*n* = 3 per group).

### 2.2. Reagents

Methyl alcohol, bovine serum albumin (BSA), TMT10plex™ (Thermo Fisher Scientific Co., Ltd., Waltham, MA, USA) isobaric label reagent set, and acetonitrile (≥99.0%) were purchased from Thermo Fisher Scientific Co., Ltd. (Waltham, MA, USA). 2-chlorophenylalanine, trichloroacetic acid, β-mercaptoethanol, and acetone were obtained from Aladdin Co., Ltd. (Los Angeles, CA, USA). Formic acid (LC-MS level) was obtained from Tokyo Chemical Industry (TCI) Co., Ltd. (Tokyo, Japan). Ammonium formate, ammonium bicarbonate, urophilia, dithiothreitol (DTT), iodoacetamide (IAM), and TEAB were purchased from Sigma-Aldrich Co., Ltd. (St. Louis, MO, USA). ZipTip and dd H_2_O were purchased from Millipore Co., Ltd. (Burlington, MA, USA). A protein quantitative dye solution was obtained from Huaxingbio Co., Ltd. (Beijing, China). Tryptase was obtained from Promega Co., Ltd. (Madison, WI, USA).

### 2.3. Color Measurement

The exterior color of the coffee cherry samples (*n* = 20 per group) was determined using a colorimeter (NR10QC+, Shenzhen 3nh Technology Co., Ltd., Shenzhen, China). The lightness (L), redness (a), yellowness (b), chroma (c), hue angle (h°), and total chromatism (ΔE) were measured at two surfaces of each coffee cherry.

### 2.4. Untargeted Metabolomics Analysis

The untargeted metabolomics analysis was carried out following the protocol described by Tian et al. [16]. Briefly, six whole coffee cherries from each group were selected and ground at low temperatures with liquid nitrogen. A total of 200 ± 2 mg of each sample was homogenized in advance by a KZ-II tissue grinder (Servicebio Co., Ltd., Wuhan, China) and poured into a 2 mL Eppendorf (EP) tube. Then, 0.6 mL of 2-chlorophenylalanine (4 parts per million, PPM) in methanol was accurately added at −20 °C and vortexed for 30 s. Following this, 100 mg glass beads were added to an EP tube, and the samples were ground at 50 Hz for 90 s. Furthermore, the samples were placed in an ultrasonic bath at room temperature for 15 min and centrifuged at 12,000× *g* for 10 min. The supernatant (300 μL) was filtered through a 0.22 μm membrane and transferred to a vial for on-machine detection by triple quadrupole LC-MS/MS. For quality assurance purposes, 20 μL of each group of samples was mixed with a quality control sample (QC). A total of 5 QC samples were obtained using the same method for further analysis.

The metabolomics analysis of the coffee cherries was carried out using a Q Exactive Plus HPLC-MS/MS system (Thermo Fisher Scientific, Waltham, MA, USA). An Acquity UPLC HSS T3 column (150 mm × 2.1 mm, 1.8 μm, Waters, Manchester, UK) was employed for analyte separation. Gradient elution was carried out with 0.1% (*v*/*v*) formic acid in water (C) and 0.1% (*v*/*v*) formic acid in acetonitrile (D) in the positive model and 5 mmol/L ammonium formate in water (A) and acetonitrile (B) at a flow rate of 0.25 mL/min in the negative model. Following equilibration of the instrument, the coffee cherry sample extracts were injected (2 μL). Gradient elution was set as follows for the mobile phase: 0–1 min, 2% B/D; 1–9 min, 2–50% B/D; 9–12 min, 50–98% B/D; 12–13.5 min, 98% B/D; 13.5–14 min, 98–2% B/D; and 14–20 min, 2% D-positive model (14–17 min, 2% B-negative model). Full-scan data were acquired between the mass range of *m*/*z* 81–1000. The positive ion spray voltage was 4.20 kV; the negative ion spray voltage was 3.50 kV; and the sheath and auxiliary gas flows were 30 arb and 10 arb, respectively. The capillary temperature and collision voltages were 325 °C and 30 eV. Moreover, dynamic exclusion was used to remove unnecessary MS/MS information. A base peak chromatogram of a typical sample is presented in Supplementary Figure S1.

The raw data were pre-treated using Proteowizard software (v3.0.8789; State of California, USA, Cedars-Sinai Medical Center) and R (v3.3.2; Auckland, New Zealand, Ross Ihaka and Robert Gentleman). Furthermore, three-dimensional data sets were obtained, including *m*/*z*, retention time (RT), and intensities of the peaks. The RT–*m*/*z* pairs were used as the identifier for each ion [17]. For a better comparison, the peak area was normalized, and any peaks with missing values (ion intensity = 0) and pseudo-positive peaks were removed. Peaks with poor repeatability in the QC samples were deleted. The proportion of potential characteristic peaks with a relative standard deviation (RSD) was less than 30% in the QC samples, and more than 70% of the peaks demonstrated high quality (Supplementary Figure S2). The metabolite pathways were subsequently mapped by the online Kyoto encyclopedia of genes and genomes (KEGG) database [17].

### 2.5. Tandem Mass Tag (TMT) Labelling Proteomics Analysis

In this section, TMT labeling proteomics analysis was also carried out according to the procedure described by Tian et al. [16]. Three whole coffee cherries from each group were randomly selected and ground with liquid nitrogen. The samples were transferred into a 6 mL extraction solution (lysis solution/protease inhibitor = 50:1 *v*/*v*) containing 5 g of trichloroacetic acid and 35 μL of β-mercaptoethanol in four volumes of cold acetone for protein extraction. After incubating overnight at −20 °C, the protein precipitate was washed with 1 mL of acetone and collected by centrifugation at 14,000× *g* for 20 min at 4 °C. Furthermore, the protein concentration was determined with the Bradford method and exhibited specific protein profiles on an SDS-PAGE (Supplementary Figure S3). As previously reported, approximately 1 mg of each protein sample was digested overnight with 20 μg of trypsin at 37 °C [18]. After digestion, the peptides were desalted by a Strata X C18 SPE column, reconstituted in 0.5 mol/L TEAB, and processed according to the manufacturer’s protocol for the TMT kit.

The peptides were dissolved in the mobile phase A of LC (0.1% formic acid) and separated using a RIGOL L-3000 HPLC system. Mobile phase A contained 0.1% formic acid, and mobile phase B was an aqueous solution containing 0.1% formic acid and 80% acetonitrile. The LC elution settings were 0–49 min, 7–40% B; 49–60 min, 100% B with 0.7 mL/min flow. The resulting peptides were subjected to tandem MS/MS after ionization of the Nanospray FlexTM (NSI) ion source on a Q Exactive HF-X mass spectrometer. The spray voltage was set to 2.4 kV, and the ion transfer tube temperature was set to 275 °C. The scanning range of the primary mass spectrum was set to 407–1500 *m*/*z*. The top 40 parent ions were selected for MS/MS with an automatic gain control (AGC) target of 3 × 10^6^. The secondary MS resolution was set at 45,000 (200 *m*/*z*) with an AGC target of 5 × 10^4^, and the maximum injection time was set at 86 ms. The normalized collision energy of 32% was used for the peptides chosen for MS/MS. The protein was quantified by using the razor and unique peptides.

The protein identification in this study was carried out using the protein database of *Coffea Arabica* L. in https://www.uniprot.org/ (accessed on 11 January 2021). The PDQuest 2.4 software (Bio-Rad Co., Ltd., Hercules, CA, USA) was used to search the database. Thermo Proteome Discoverer software version 1.4 (Thermo Fisher Scientific, Bremen, Germany) was used with the default settings to process the raw data. According to those reported in the UniProt database, the gene ontology (GO) annotations were performed for the identified proteins. A KEGG enrichment-pathway analysis was further performed for the identified proteins.

### 2.6. Statistics

Data analysis was carried out using SPSS Version 29.0 (IBM Corp., New York, NY, USA), R (v3.3.2) and GraphPad Prism 9 (GraphPad Software Inc., San Diego, CA, USA). One-way analysis of variance (ANOVA) followed by Duncan’s post hoc test was carried out to perform multi-group comparisons. *p* < 0.05 was considered statistically significant. “**” represents the significant differences (*p* < 0.01). Multivariate statistical analysis was carried out using MetaboAnalyst Version 5.0 (Xia-lab, Montreal, QC, Canada).

## 3. Results and Discussion

### 3.1. Changes in Coffee Cherry Skin Color

**Figure 1:**
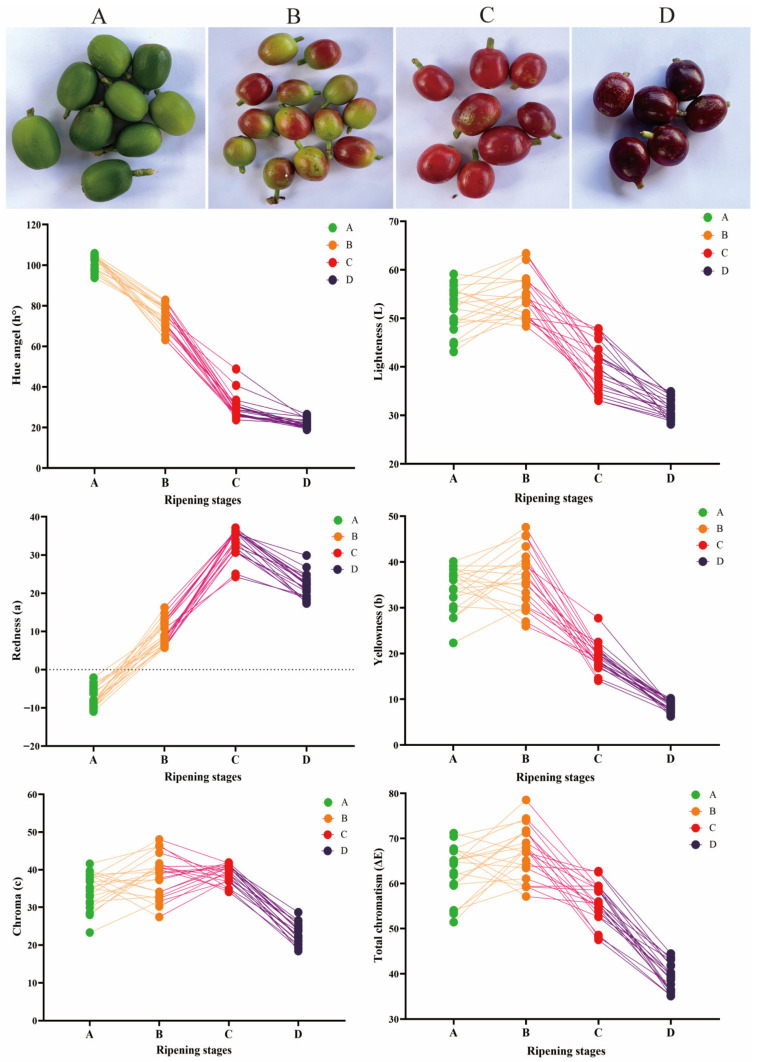
Changes in skin color during coffee cherry ripening.

Fruit color indicates maturity level and quality stage and is an essential parameter in fruit classification [19]. As the coffee cherry grows in different stages of maturity, changes in skin color would be observed, and flavor precursors will accumulate. Coffee maturity was divided into A–D ripening stages (Figure 1), and the color of the coffee cherries was presented as green–brown–red–deep red, respectively. Moreover, the values of a, b, c, h°, and (E) demonstrated changes at different ripening stages. Specifically, an “a” value (redness) of −7.37 ± 2.54 was below 0 in the A group, indicating that these samples had a primarily green color. However, the “a” value of group C was 33.43 ± 3.60, suggesting that the coffee cherries ripened from having green skin to red skin. Coffee cherry maturity was attributed to pigments accumulating outside the skin and the changing components in the inner tissue [20].

### 3.2. Overview of Untargeted Metabolomics

**Figure 2:**
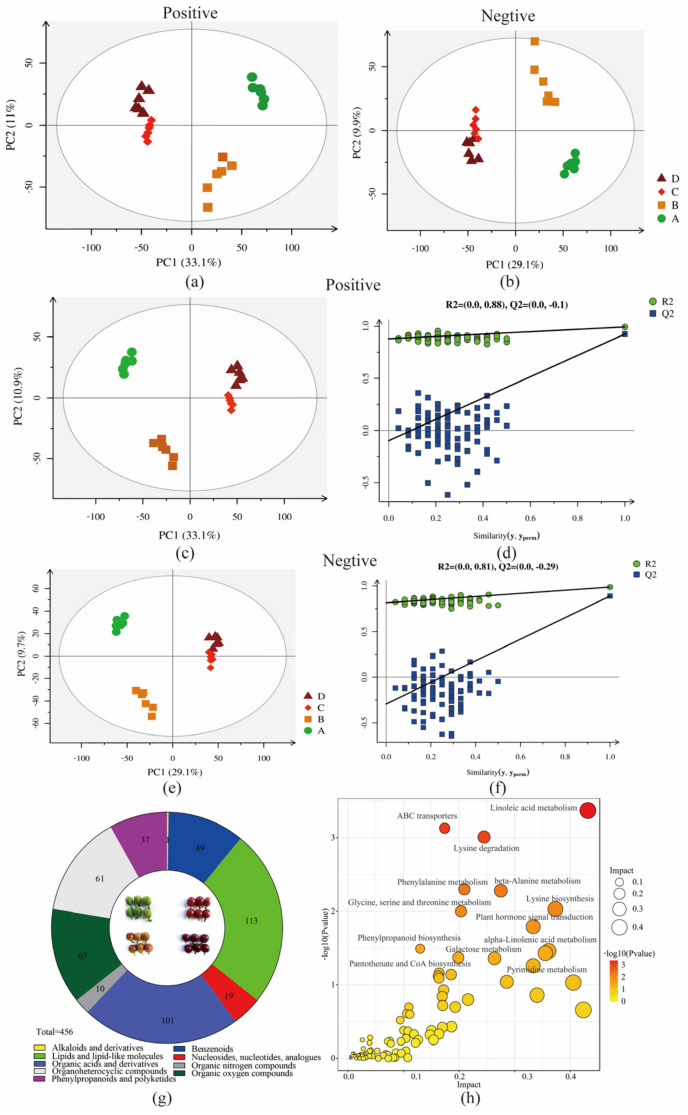
Principal component analysis (PCA) of peak areas detected in four groups of coffee samples in positive and negative ion models (**a**,**b**) (four maturity stages: A (●), B (■), C (◆), D (▲)). Partial least squares discriminant analysis (PLS-DA) and permutation test of peak areas in positive and negative ion models in four group samples (**c**–**f**). Classification of DEMs in four group samples (**g**). KEGG pathways of DEMs involved (**h**).

There were 10,807 and 8376 detectable peaks in the positive and negative modes, respectively (Supplementary Data S1, sheets 1–2, and Supplementary Figure S1). In the positive and negative models, the QC samples gathered together, indicating that the data had few errors and higher quality. Furthermore, a total of 70.5% and 88.8% of the peaks with RSD less than 30% were in the positive and negative ion modes, respectively, which also well proved the high data quality (Supplementary Figure S2). After raw data pre-treatment, the positive (7618) and negative (7435) data were combined into a data set (Supplementary Data S1, sheet 3–4) and were imported into the R package (v3.2.2). Furthermore, a principal component analysis (PCA) and a partial least squares discriminant analysis (PLS-DA) were performed to visualize the metabolic differences among the samples from the four maturity stages. The differential clustering among the four stages of coffee cherries in the PCA (Figure 2a,b) and PLS-DA (Figure 2c,e) obtained similar results. The samples were separated into groups, and aggregation of the same group indicated that there were differences in the metabolite profiles at each maturity stage but similar metabolites at the same maturity stage. A permutation test was conducted to verify whether the PLS-DA model of the data had been over-fitted. The results showed that all blue Q2 points were lower than the original from left to right (Figure 2d,f). These results suggested that different maturity stages of coffee cherries had distinctly different metabolite characteristics.

Based on MS/MS spectra, retention time, and metabolomics database, 2178 nonvolatile compounds were identified (Supplementary Data S1, sheet 5). Then, a variable importance in projection (VIP) analysis ranked the overall contribution of each variable to the PLS-DA model, and variables with VIP > 1.0 and *p* < 0.05 were classified as difference express metabolites (DEMs). Finally, a total of 456 DEMs were selected, and they were identified as being concentrated in the four maturity stages of coffee cherries, such as glucose, sucrose, tyrosine, cystathionine, palmitic acid, and quercetin. These DEMs were divided into super-classes of alkaloids and derivatives (trigonelline), benzenoids (49 metabolites), lipids and lipid-like molecules (113 metabolites), nucleosides, nucleotides and analogs (19 metabolites), organic acids and derivatives (101 metabolites), organic nitrogen compounds (10 metabolites), organic oxygen compounds (61 metabolites), organoheterocyclic compounds (65 metabolites), and phenylpropanoids and polyketides (37 metabolites) (Supplementary Data S1, sheet 6, and Figure 2g). DEMs were further grouped into different classes, including flavonoids, sugars, amino acids, and fatty acids. These DEMs were significantly enriched into 87 KEGG pathways such as linoleic acid metabolism, lysine biosynthesis, galactose metabolism, and flavonoid biosynthesis (Supplementary Data S1, sheet 7, and Figure 2h). Some DEMs were closely related to the characteristic flavor formation as important precursors during coffee cherry ripening. For example, mature coffee cherries could produce more concentrations of carbohydrate and aroma compounds of carbohydrate degradation with higher sensory quality scores than immature green cherries [21].

### 3.3. Overview Analysis of TMT Labelling Proteomics

**Figure 3:**
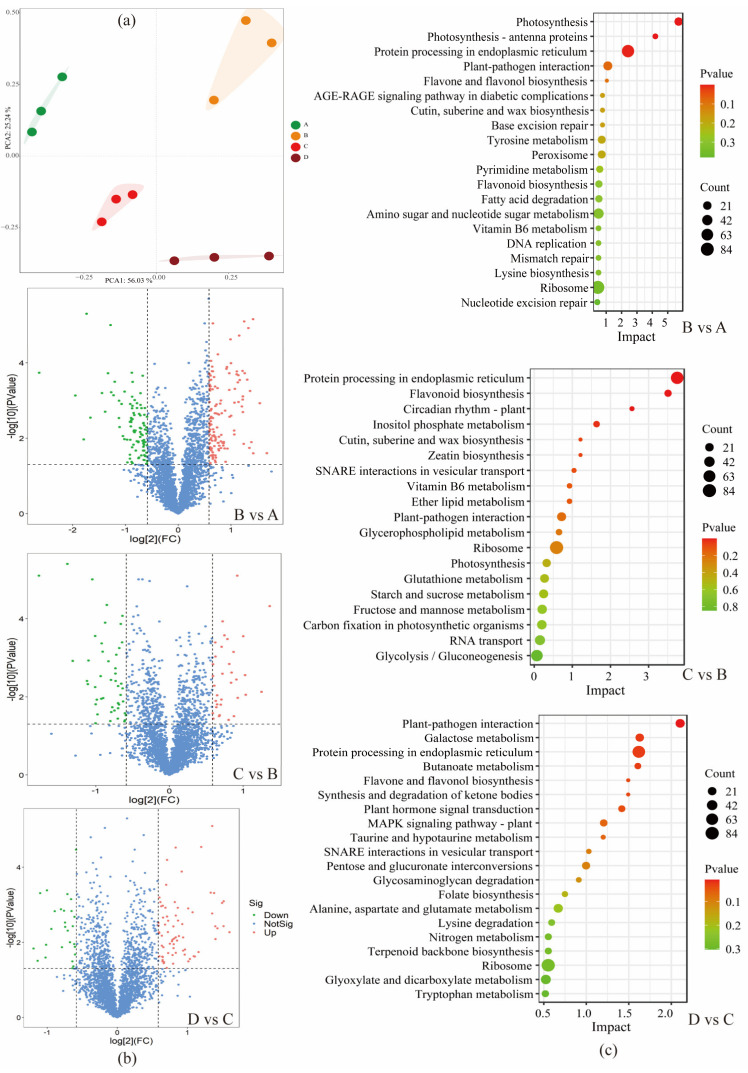
Results of proteomics overview analysis. PCA score of four coffee sample groups (**a**). Volcanic map analysis of DEPs. Red: up-regulation; green: down-regulation; blue: not significant. A total of 125, 34, and 71 proteins were up-regulated, while 98, 47, and 27 proteins were down-regulated (**b**). The enrichment analysis of the KEGG pathway in coffee at each maturation level and the top 20 pathways in each stage (**c**).

Previous research observed that the metabolites expressed generally fall behind alterations in corresponding proteins, indicating that protein expression changes are vital factors in triggering metabolic changes [22]. A TMT labeling proteomics analysis was employed to reveal the underlying molecular mechanism for the changes in nonvolatile compounds during the different maturity stages of coffee cherries. A total of 11,309 peptides were obtained (Supplementary Data S2, sheet 1), and the length of the peptides ranged mainly from 14 to 26 (Supplementary Figure S4a). The range of the peptide-spectrum match (PSM) captured per peptide segment with a mass spectrum ranged between 1 and 5 (Supplementary Figure S4b), indicating that the lengths of the peptides were reasonable, with good accuracy and reliability for identifying peptides. The molecular weights of most identified proteins ranged from 10 to 100 kDa (Supplementary Figure S4c), suggesting that the proteins have a reasonable molecular weight. Furthermore, these peptides were matched with 2748 unique proteins in the samples from the four maturity stages. The proteins included beta-galactosidase (A0A6P6WN40), cysteine protease (A0A6P6W856), and aminomethyltransferase (A0A6P6U842) (Supplementary Data S2, sheet 2). The results of the PCA showed that the samples from the four maturity stages were clustered together in different clusters (Figure 3a). Furthermore, the condition of FC ≥ 1.5 and *p* < 0.05 were employed to select the expressed proteins (DEPs). A total of 223 (B vs. A), 81 (C vs. B), and 98 (D vs. C) DEPs were identified, including carbohydrate synthetic protease, amino acid synthetic protease, lipase, and enzymes related to the synthesis of flavonoid polyphenols (Supplementary Data S2, sheet 3–5). There were 125 (B vs. A), 34 (C vs. B), and 71 (D vs. C) proteins up-regulated and 98 (B vs. A), 47 (C vs. B), and 27 (D vs. C) proteins down-regulated (Figure 3b).

The highly represented GO classes of DEPs include the molecular function (molecular level activities such as catalytic or binding), the biological process (cellular and metabolic processes), and the cellular component (cell part and its extracellular environment) (Supplementary Figure S5). The 102 terms of the KEGG pathways were enriched in the four stages of coffee cherries, with metabolic pathways and biosynthesis being the most enriched terms. These enrichments of the KEGG pathways included amino sugar and nucleotide sugar metabolism, glutathione metabolism, photosynthesis, flavonoid biosynthesis, fructose and mannose metabolism, glycolysis/gluconeogenesis, galactose metabolism, inositol phosphate metabolism, and fatty acid degradation (Supplementary Data S2, sheet 6, and Figure 3c).

Because of the formation of the metabolites by enzyme regulation, most metabolites have been proven to contribute directly to flavor or to serve as flavor precursors [1,23]. For example, flavonoids could provide a bitter and astringent taste; free amino acids present an umami taste; sugar contributes to a sweet taste; and organic acids contribute to a sour taste [18]. Thus, the following analyses mainly focused on the biosynthesis and metabolism pathways of organic acids, amino acids, flavonoids, and sugars and the effect of relevant protease in the four stages of coffee cherry ripening.

### 3.4. Sugar-Related Flavor Precursors

The Maillard reaction was the main pathway for producing flavor during the roasting process, and amino acids and sugars are the most important precursors to the Maillard reaction [24]. However, different amino acids and sugars as precursors could generate different coffee flavors. A high sugar content not only increases the sweetness of coffee but also protects the beans from oxidation by forming a thin sugar film on the surface and facilitating the Maillard reaction [25]. Moreover, the higher sugar content (mainly sucrose, glucose, and fructose) could also increase the levels of pyrazines, furans, ketones, and heterocyclic nitrogen-containing compounds after roasting. These compounds mainly contribute to the flower, fruit, and caramel roast aroma of coffee [25].

**Figure 4:**
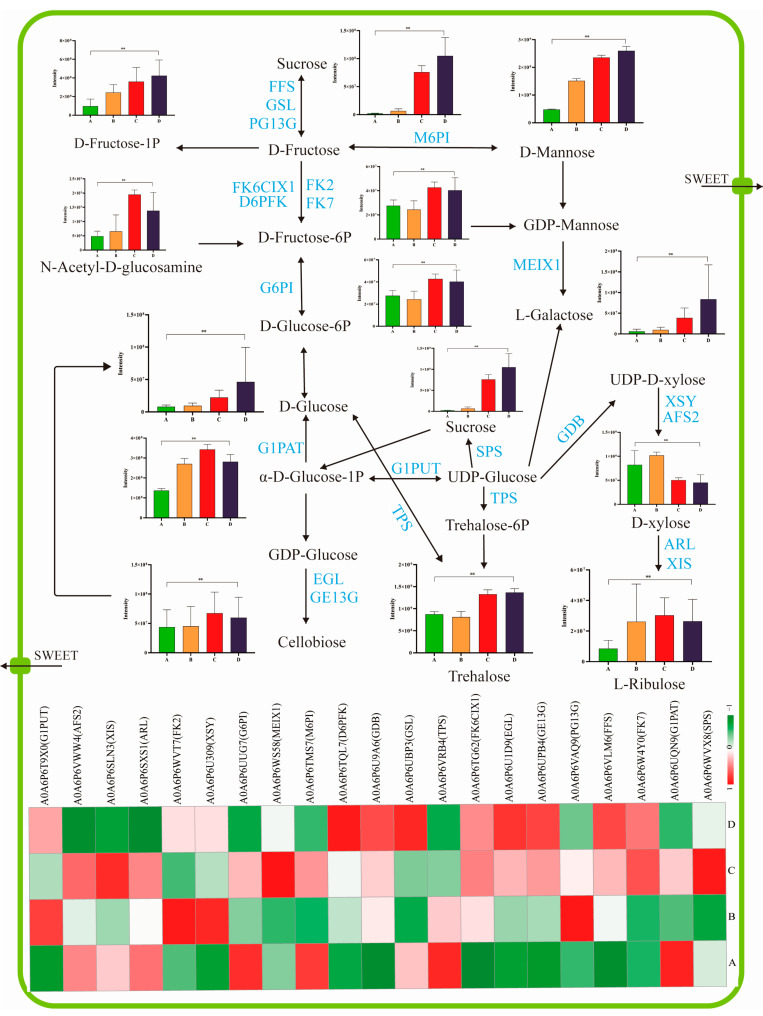
Pathway viewer of main sugar metabolism in coffee cherries. The ripening progression of protein expression from A to D is presented in four box strings. “**” represents the significant differences (*p* < 0.01). Putative protein families were from the *Coffea Arabica* L. genome database. G1PUT, UTP-glucose-1-phosphate uridylyltransferase; AFS2, α-L-arabinofuranosidase 2; XIS, xylose isomerase; ARL, aldose reductase-like; FK2, fructokinase-2; XSY, UDP-D-xylose synthase 2; G6PI, glucose-6-phosphate isomerase; MEIX1, GDP-mannose 3,5-epimerase isoform X1; M6PI, mannose-6-phosphate isomerase; D6PFK, ATP-dependent 6-phosphofructokinase; GDB, UDP-glucuronate decarboxylase; GSL, α-glucosidase-like; TPS, α,α-trehalose-phosphate synthase; FK6CIX1, fructokinase-6, chloroplastic isoform X1; EGL, endoglucanase; GE13G, glucan endo-1,3-beta-D-glucosidase; FFS, β-fructofuranosidase; FK7, fructokinase-7; G1PAT, glucose-1-phosphate adenylyltransferase; SPS, sucrose-phosphate synthase.

The sugar contents of coffee cherries gradually increase as cherries mature, especially the content of fructose, sucrose, and galactose, reaching 15%, 3.2%, and 2.4%, respectively [26]. In this study, the contents of sucrose, fructose, glucose, mannose, trehalose, and galactose markedly increased from ripening stages A to D. During the early maturity stages, the concentrations of cellobiose, xylose, and ribulose significantly increased. However, the level of these sugars decreased at a later maturity stage (Figure 4). In addition, 21 important DEPs related to the biosynthesis and degradation of sugars were identified in this study. A previous study showed that sucrose is simultaneously regulated by synthetic and degradative enzymes during fruit ripening and potentially degraded to fructose through β-fructofuranosidase regulation [27]. Our proteomic results showed that the level of β-fructofuranosidase (A0A6P6VLM6, FFS) and α-glucosidase (A0A6P6UBP3, GSL) showed a positive relationship with sucrose and fructose (Figure 4), indicating that these enzymes lead to the accumulation of the two reducing sugars during the ripening process. However, the level of glucan 1,3-α-glucosidase (A0A6P6VAQ9, PG13G) increased in the first stage. Moreover, glucan 1,3-α-glucosidase (A0A6P6VAQ9, PG13G) levels decreased with time, suggesting a negative relationship with the accumulation of sucrose and fructose. Furthermore, fructose and mannose could achieve mutual transformation by regulating with mannose-6-phosphate isomerase (A0A6P6TMS7, M6PI) [16]. Glucose was reported as a product derived from both trehalose and α-lactose and correlated with the levels of relative enzymes [16]. In this study, the expression of α,α-trehalose phosphate synthase (A0A6P6VRB4, TPS) and glucose-6-phosphate isomerase (A0A6P6UUG7, G6PI) gradually decreased (*p* < 0.05) along with glucose formation. This incidence suggests that the total glucose pool could be enlarged by utilizing the two phosphate enzymes. The activities of endoglucanase (A0A6P6U1D9, EGL) and glucan endo-1,3-β-D-glucosidase (A0A6P6UPB4, GE13G) increased at all stages, further indicating cellobiose accumulation during coffee cherry ripening.

Studies proposed that sucrose-phosphate synthase participated in the cycle of sucrose synthesis and degradation during fruit ripening [16]. In the current study, the level of sucrose-phosphate synthase (A0A6P6WVX8, SPS) was the highest at stage C (Figure 4), indicating that this stage of sucrose synthesis and degradation was very active. Based on the flavor contribution, sucrose could also become one of the critical items that could indicate the optimal harvest time for coffee cherries.

### 3.5. Amino-Acid- and Organic-Acid-Related Flavor Precursors

**Figure 5:**
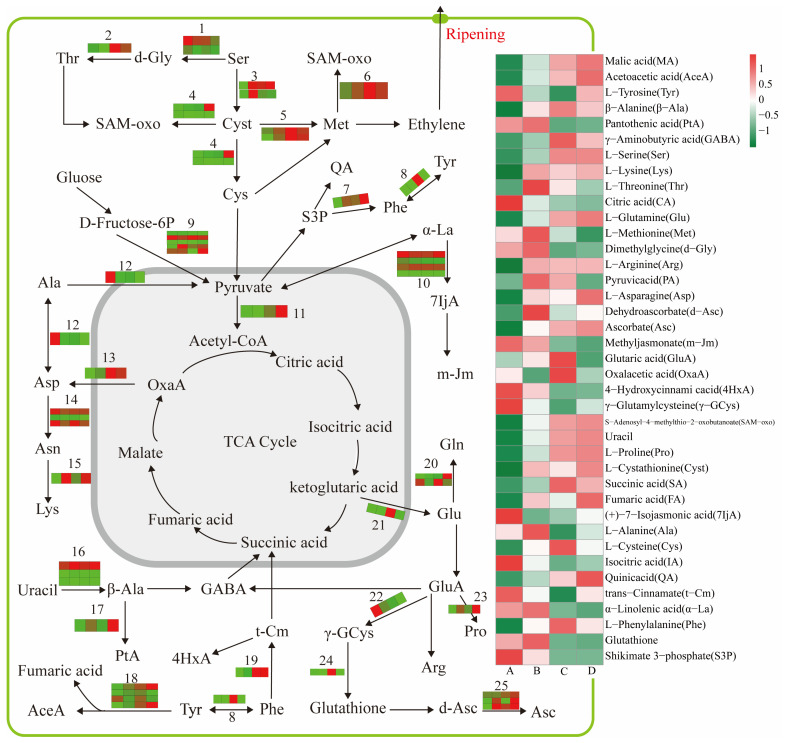
Pathway displays a subset of organic acid and amino acid metabolism proteases required in coffee cherries. The metabolites changing as ripening progresses from A to D are presented in four box strings. Heat maps, for each numbered reaction, were drawn using the TMT value. The numbered reactions were catalyzed by the following enzymes: 1, serine hydroxymethyltransferase (A0A6P6TLR0) and glutamate-glyoxylate aminotransferase 2 (A0A6P6VAJ1); 2, L-threonine aldolase 1 (A0A6P6VRS3); 3, homoserine kinase (A0A6P6VCB8) and cysteine synthase (A0A6P6WQV5); 4, L-cysteine desulfhydrase (A0A6P6VZM9) and farnesylcysteine lyase (A0A6P6T9Z7); 5, 5-methyltetrahydropteroyl triglutamate homocysteine S-methyltransferase (A0A6P6SM43); 6, S-adenosylmethionine synthase (A0A6P6SUD6); 7, shikimate dehydrogenase (A0A6P6X2E2); 8, arogenate dehydratase (A0A6P6W2X6); 9, NADP-dependent glyceraldehyde-3-phosphate dehydrogenase (A0A6P6SZ76), pyruvate phosphate dikinase (A0A6P6WRX8), phosphoglycerate mutase (A0A6P6SCM7), phosphopyruvate hydratase (A0A6P6T3Q6), and glyceraldehyde-3-phosphate dehydrogenase (A0A6P6VC84); 10, allene oxide synthase (A0A6P6UTE2), allene oxide synthase 1 (A0A6P6UPD2), allene-oxide cyclase (A0A6P6TAJ2), enoyl-CoA hydratase 2 (A0A6P6TYF9); 11, acetyl-CoA acetyltransferase (A0A6P6SED1); 12, alanine-glyoxylate transaminase (A0A6P6TM10); 13, alanine aminotransferase 2 (A0A6P6X499); 14, asparagine synthetase (A0A6P6TA86), L-asparaginase 1 (A0A6P6X331), probable L-asparaginase 3 (A0A6P6VNC7); 15, diaminopimelate decarboxylase 1 (A0A6P6VRS2); 16, dihydrothymine dehydrogenase (A0A6P6XK57), dihydropyrimidinase (A0A6P6SXN9), β-ureidopropionase (A0A6P6WDT2); 17, alanine-tRNA ligase (A0A6P6V5B4); 18, aspartate-prephenate aminotransferase (A0A6P6TCB7), tyrosine aminotransferase (A0A6P6S801), histidinol-phosphate aminotransferase (A0A6P6VSK7), fumarylacetoacetase (A0A6P6UAI6); 19, phenylalanine ammonia-lyase (A0A6P6VDX4); 20, glutamine synthetase (A0A6P6T536), glutamate synthase (A0A6P6W7J9), 21, glutamate dehydrogenase (A0A6P6SVM6); 22, γ-glutamylcyclotransferase family protein (A0A6P6UG48); 23, acetylglutamate kinase (A0A6P6TDN5); 24, glutathione synthetase (A0A6P6W069); 25, L-ascorbate peroxidase 2 (A0A6P6W0Y9), L-ascorbate peroxidase 3 (A0A6P6VST3), L-ascorbate peroxidase 6 (A0A6P6UR23).

Amino acids are among the main parameters for evaluating fruit quality. During the fruit ripening process, their levels and compositions could change substantially [28]. In addition to being precursors, amino acids could directly contribute to sweet, umami, or bitter tastes. For instance, alanine contributes to the sweetness of fruits. Aminotransferase plays a vital role in the synthesis or degradation of aspartate and pyruvic acid and could also catalyze the conversion of amino acids during fruit ripening [29]. In this study, 40 DEMs and 45 DEPs related to amino acids significantly contributed to flavor formation during coffee cherry ripening. Among them, the contents of aspartate and pyruvic acid increased but that of alanine decreased. The level of alanine-glyoxylate transaminase (A0A6P6TM10) decreased (*p* < 0.05) from stages A to D, suggesting that alanine could convert to other amino acids via the action of alanine-glyoxylate transaminase (A0A6P6TM10) (Figure 5). Cysteine, glutathione, and methionine are sulfur (S)-containing amino acids in green coffee beans and have been linked explicitly to the cooked meat flavor. This flavor has been attributed to alkyl pyrazines, diketones, 2-furfurylthiol, and other S-containing compounds decomposed by S-containing amino acids [30]. Cysteine could be synthesized by serine in the regulation of homoserine kinase and cysteine synthase, and methionine was negatively confirmed with the ethylene biosynthesis during fruit ripening [31,32]. In the present study, serine, cysteine, and cystathionine increased, while glutathione, methionine, and γ-glutamylcysteine decreased during coffee cherry ripening. The level of relevant proteases, including homoserine kinase (A0A6P6VCB8), cysteine synthase (A0A6P6WQV5), L-cysteine desulfhydrase (A0A6P6VZM9), farnesyl cysteine lyase (A0A6P6T9Z7), 5-methyl-tetrahydro pteroyl triglutamate homocysteine S-methyltransferase (A0A6P6SM43), S-adenosylmethionine synthase (A0A6P6SUD6), and glutathione synthetase (A0A6P6W069), were also upregulated (Figure 5). This result suggested that the above proteases regulated S-containing amino acid biosynthesis in coffee. The decrease in the content of methionine implied an ethylene biosynthesis increase, which was also connected to ripening and consistent with the findings of a previous study [32]. In addition, the amount of β-alanine, glutamine, phenylalanine, and the relevant enzymes increased during coffee cherry ripening. These constituents, which acted as precursors, participated in organic acid biosynthesis.

The organic acid fraction in green coffee is composed of volatile and nonvolatile aliphatic (citric, malic, and quinic acid) and phenolic acids (about 8%) [33]. These acids were highly relevant for the acidic taste in the cherries. The nonvolatile organic acids could serve as a precursor to form volatile phenols such as 2-methoxy phenol (guaiacol), 4-ethyl-2-methoxy phenol (4-ethyl guaiacol), and 4-vinyl-2-methoxy phenol (4-vinyl guaiacol). These compounds potentially provide the typical smoky and woody aroma in dark roasted coffee beans [34,35]. The tricarboxylic acid cycle (TCA) is a vital biological process involved in the biosynthesis and derivatization of organic acids [36]. Pyruvic acid is a critical precursor of the TCA cycle and a junction among sugars, organic acids, and amino acids, and its degradation is accompanied by a series of conversions between organic acids to meet the needs for intermediates and energy during fruit ripening [37,38]. Among metabolites in the TCA cycle, fumaric acid, malic acid, and succinic acid increased at all stages. On the other hand, pyruvic acid, citric acid, and isocitric acid declined (Figure 5), indicating that these organic acids dynamically changed during the ripening process.

### 3.6. Flavonoid-Related Flavor Precursors

**Figure 6:**
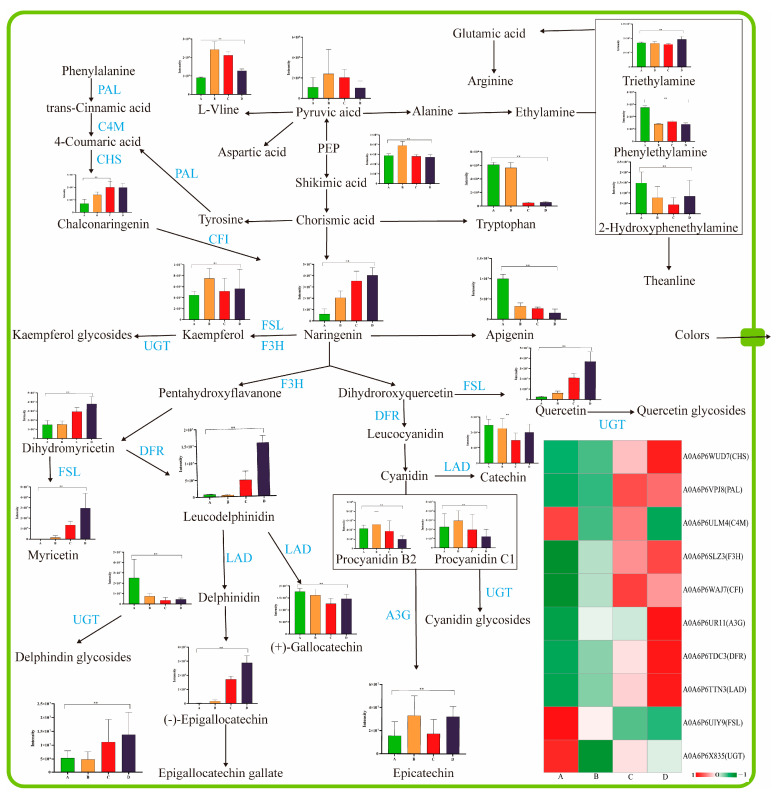
Diagram of phenylpropanoids and flavonoids biosynthesis pathways in the coffee cherries at different maturity stages. The ripening progression of protein expression from A to D is indicated in four box strings. “**” represents the significant differences (*p* < 0.01). CHS, chalcone synthase; PAL, phenylalanine ammonia-lyase; C4M, trans cinnamate 4-monooxygenase; F3H, flavanone 3-hydroxylase; CFI, chalcone flavanone isomerase; A3G, anthocyanidin 3-O-glucosyltransferase; DFR, dihydroflavonol 4-reductase; LAD, leucoanthocyanidin dioxygenase; FSL, flavonol synthase-like.

Flavonoids, especially flavonols, contribute tremendously to the astringent taste, color, and aroma formation of fruits. The phenylpropanoid and flavonoid biosynthesis pathways were principally for the biosynthesis of flavonoids [18,39]. During fruit ripening, there was a dramatic change in flavonoid composition and content, which was associated with the transformation of fruit pigmentation and flavor [40]. In this study, 10 DEPs were annotated as crucial enzymes involved in the biosynthesis of phenylpropanoids and flavonoids. These levels of enzymes were upregulated besides trans-cinnamate 4-monooxygenase (A0A6P6ULM4, C4M), flavonol synthase-like (A0A6P6UIY9, FSL), and flavanol 3-O-glucosyltransferase (A0A6P6X835, UGT) during Arabica coffee cherry ripening (Figure 6). As the first rate-limiting enzyme in the flavanol biosynthesis pathway, phenylalanine ammonia-lyase directly catalyzes the degradation of phenylalanine to trans-cinnamic acid [18]. Phenylalanine ammonia-lyase (A0A6P6VPJ8, PAL) and phenylalanine were upregulated, and trans-cinnamic acid was downregulated. This result indicated that trans-cinnamic acid was the substrate that could synthesize chalcone and naringenin by combining with trans-cinnamate 4-monooxygenase (A0A6P6ULM4, C4M), chalcone synthase (A0A6P6WUD7, CHS), and chalcone-flavanone isomerase family protein (A0A6P6WAJ7, CFI) in the phenylpropanoids pathway. In addition, the content of naringenin and chalconaringenin continuously increased from stages A to D (Figure 5 and Figure 6). These results further confirmed the above inference. The naringenin further decomposed and was involved in the synthesis of other flavonoids in the flavonoids biosynthesis pathway. Considering the nutrition and the astringent taste of flavonoids, maturity stage C could be considered a suitable harvest stage when flavor precursors were prepared. In addition, glycosidic compounds were also vital in green coffee beans because the aglycones could be released during the roasting process to enrich the aroma profile [41]. Cyanidin glycosides (*n* = 5), delphinidin glucosides (*n* = 2), kaempferol glycosides (*n* = 5), and quercetin glycosides (*n* = 2) were detected in this study. These glycosides exhibited various expression trends during ripening, mainly quercetin 3-O-glucoside, which possessed the highest ionic peak intensity at all four stages (Figure 6 and Supplementary Data S2, sheet 2).

In summary, sugar, amino acids, and organic acids metabolism in fresh coffee cherries could contribute to the flower, fruit, and caramel roast aroma and sweet taste as a flavor candidate. The flavonoid fraction could contribute tremendously to the astringent taste and skin color. In addition, the environment, such as altitude, soil, daylight hours, and precipitation, is also a potential factor that could affect the ripening of fresh coffee cherries. For instance, coffee cherry has a higher sensory quality when it ripens a month later at higher altitudes than at lower altitudes, [42,43]. The coffee cherry flavor is directly linked to the chemical composition of the raw bean and ultimately affects the flavor property of the coffee product. These results can complement knowledge of the formation of coffee flavor precursors during the ripening process and can increase the understanding of coffee flavor. However, this study design was based on coffee cherry’s four maturity stages, which were divided based on coffee cherry skin color, and then, proteomics and metabolomics profiles were integrated to comprehensively investigate the flavor precursor involved in Arabica coffee cherry ripening. If the maturity stages of coffee cherry are evaluated by other sensory analysis indices, such as hardness, the difference express metabolites and crucial enzymes that contribute to flavor precursors formation might be slightly different. A future study will be attempted to evaluate coffee cherry flavor precursor changes during Arabica coffee cherry ripening based on the total sensory quality properties of cherries.

## 4. Conclusions

In the present study, the coffee cherry skin colors mainly presented green–brown–red–deep red changes from the A to D ripening stages, as detected by the NR10QC+ colorimeter. A comprehensive analysis of *coffea arabica* L. cherries integrating metabolomics and proteomics was performed on a large scale in whole cherries to understand the dynamic changes in metabolites and proteins that regulate the content of the flavor precursor during the maturity stage of the coffee. The metabolomics and proteomic results identified 456 common DEMs and 223 (B vs. A), 81 (C vs. B), and 98 (D vs. C) DEPs, respectively. Furthermore, the critical precursor metabolites and the relevant enzymes (especially amino acids and organic acids metabolism, flavonoid biosynthesis, and sugar metabolism) with their associated pathways were confirmed. Our results provide an integrated view on the significant pathways about the functions of metabolites and proteins required for coffee cherry flavor precursors and new insights into the processes underlying the fruit ripening traits.

## Data Availability

The data are contained within the article and the Supplemental Data, and the data presented in this study are available from the corresponding author upon request.

## Supplementary Materials

The following supporting information can be downloaded at https://www.mdpi.com/article/10.3390/foods12071432/s1, Supplementary Data S1: Original data of metabolomics; Supplementary Data S2: Original data of proteomics; Figure S1: Base peak chromatogram of typical sample in metabolomics; Figure S2: QC and RSD of coffee cherries metabolomics; Figure S3: SDS-page of coffee cherry protein in proteomics; Figure S4: Length distribution, special PSM numbers, and Mw of peptide of coffee cherries in proteomics; Figure S5: GO terms of DEPs in proteomics.
