# Supplementary material for: Integrating Metabolomics and Proteomics Technologies Provides Insights into the Flavor Precursor Changes at Different Maturity Stages of Arabica Coffee Cherries
Source: Foods. 2023 Mar 28;12(7):1432. doi: 10.3390/foods12071432 (PMC10094060; doi:10.3390/foods12071432)
Supplement: Supplementary file 1 [file foods-12-01432-s001.zip › foods-2272154-supplementary.pdf]

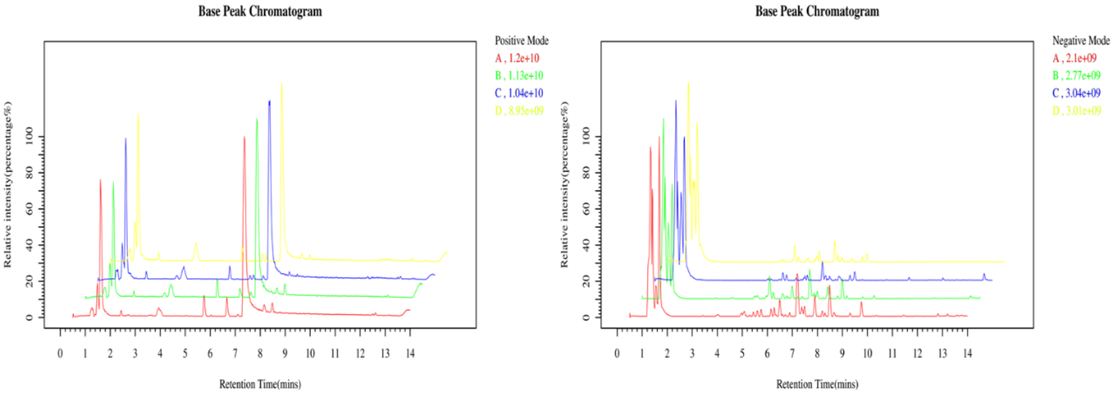

Supplementary Figure S1. Base peak chromatogram of typical sample in metabolomics.

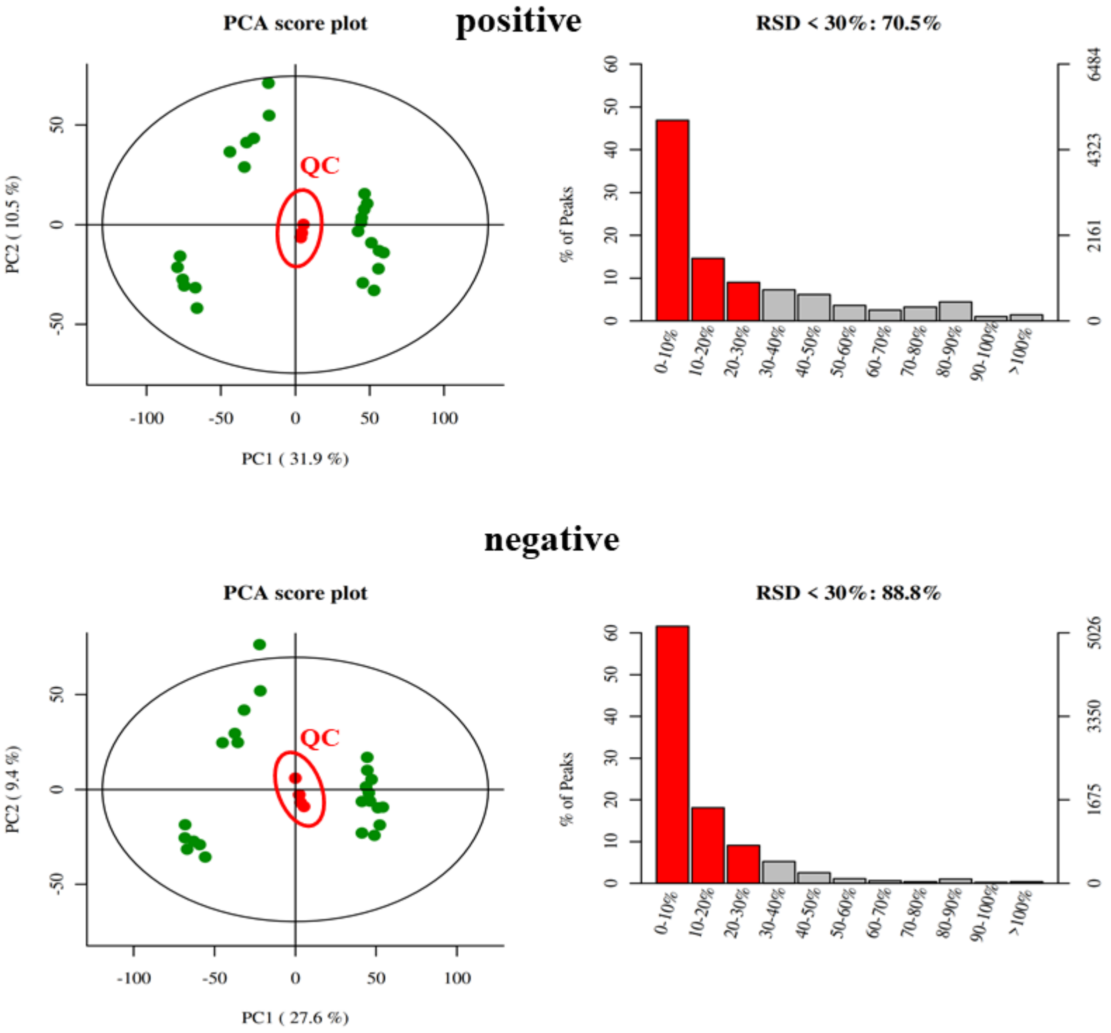

Supplementary Figure S2. QC and RSD of coffee cherries metabolomics.

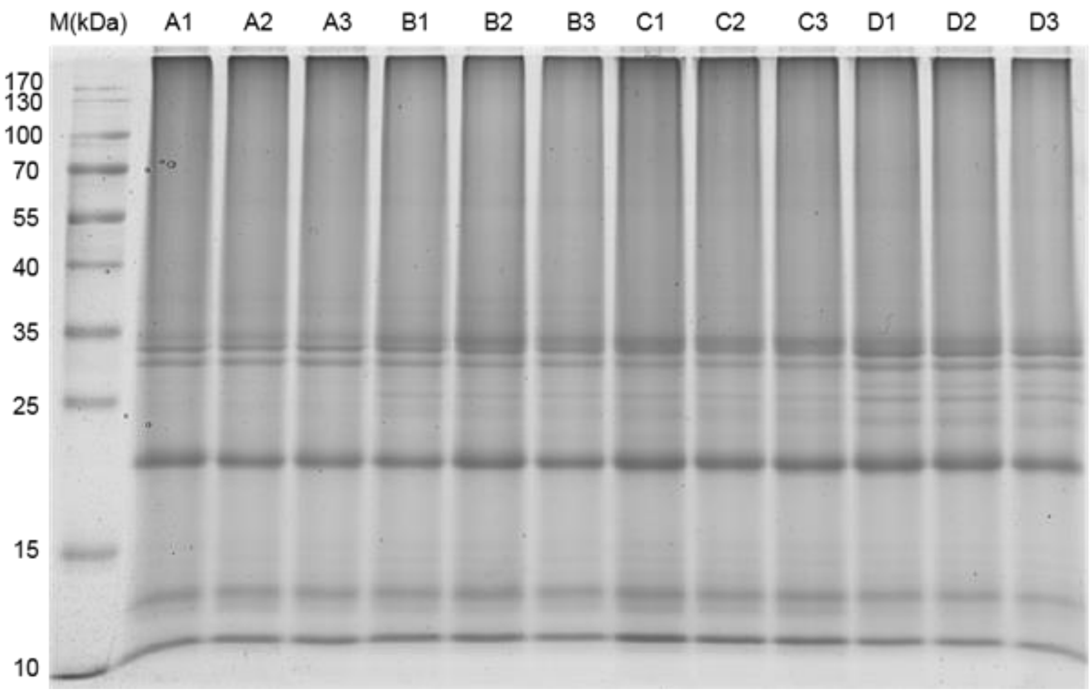

Supplementary Figure S3. SDS-page of coffee cherry protein in proteomics.

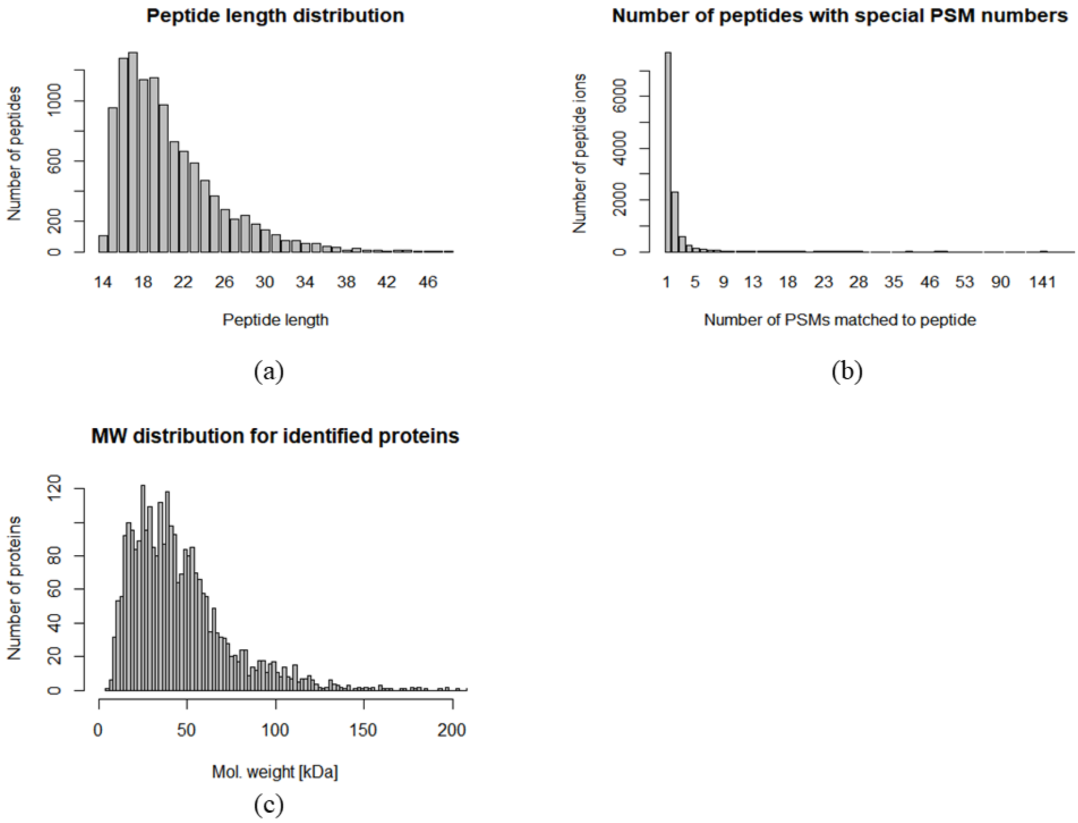

Supplementary Figure S4. Length distribution, special PSM numbers, and Mw of peptide of coffee cherries in proteomics.

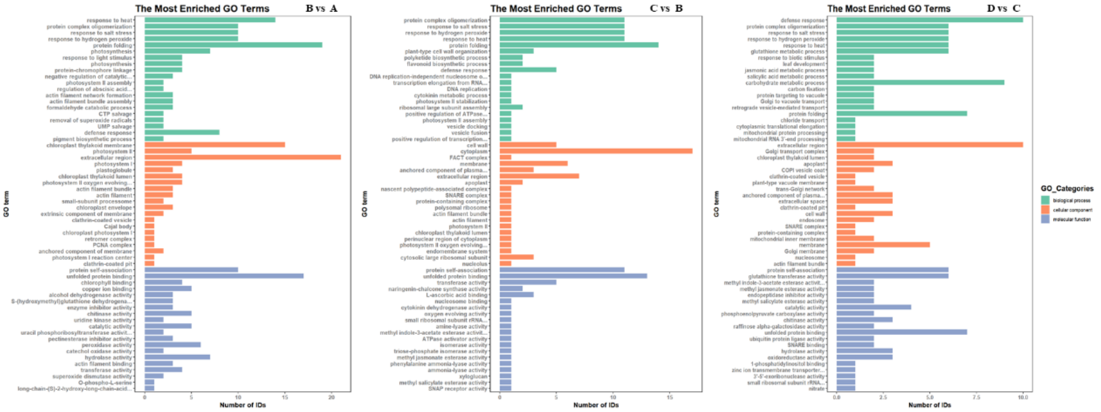

Supplementary Figure S5. GO terms of DEPs in proteomics.
